# The Impact of Alcohol Sales in A College Football Stadium on Healthcare Utilization

**DOI:** 10.5811/westjem.2022.11.58766

**Published:** 2023-02-25

**Authors:** David Ruehlmann, Christopher Halbur, Cassandra Moylan, Peter Georgakakos, Matthew Negaard, Hans House

**Affiliations:** * University of Iowa, Department of Emergency Medicine, Iowa City, Iowa; † University of Iowa, Carver College of Medicine, Iowa City, Iowa

## Abstract

**Introduction:**

In 2021, a large Midwestern university began selling alcohol to spectators within the football stadium for the first time. The stadium routinely hosts >65,000 spectators, and drinking alcohol is highly prevalent at pre-game tailgating events. Our goal in this study was to determine the impact of in-stadium alcohol sales on the incidence of alcohol-related emergency department (ED) visits and local emergency medical services (EMS) calls. We hypothesized that the availability of alcohol throughout the stadium would lead to an increase in alcohol-related patient presentations.

**Methods:**

This was a retrospective study including patients who used local EMS and presented to the ED on football Saturdays in the 2019 and 2021 seasons. There were 11 Saturday games with seven home games each year. The 2020 season was excluded due to the impact of COVID-19- related restrictions on attendance. Trained extractors using predefined criteria reviewed records for each patient to determine whether the visit was alcohol related. Using logistic regression analysis we examined the odds of an EMS call and ED visit being alcohol-related before and after the start of stadium alcohol sales. We compared characteristics of visits before and after the onset of stadium alcohol sales using Student’s t-test for continuous variables and chi-square test for categorical variables.

**Results:**

In 2021, after the onset of in-stadium alcohol sales, there were a total of 505 emergency calls to local EMS on football Saturdays (home and away), and 29% of them were for alcohol-related incidents down from 36% of 456 calls in 2019. After adjustment for covariates, the odds of a call being alcohol-related were lower in 2021 than 2019, but this difference was not significant (adjusted odds ratio [aOR] 0.83, 95% CI 0.48–1.42). Looking specifically at the seven home games each season, the difference was more pronounced (31% of calls in 2021 compared to 40% in 2019) but not statistically significant after adjustment for covariates (aOR 0.54, 95% CI 0.15–2.03). In the ED, 1,414 patients were evaluated on game days in 2021 and 8% of them for alcohol-related reasons. This is similar to 2019, when 9% of the 1,538 patients presented due to alcohol-related complaints. After adjustment for covariates, the odds of an ED visit being alcohol-related were similar in 2021 and 2019 (aOR 0.98, 95% CI 0.70–1.38).

**Conclusion:**

There was a decrease in alcohol-related EMS calls on home game days in 2021, although the result was not statistically significant. In-stadium alcohol sales had no significant impact on the frequency or proportion of alcohol-related ED visits. The reason for this outcome is unclear, but it is possible that fans drank less at tailgate parties knowing they could consume more once the game started. Long lines and a two-beverage limit at stadium concessions may have kept patrons from consuming excessively. The results of this study may inform similar institutions regarding the safe implementation of alcohol sales during mass-gathering events.

## INTRODUCTION

Mass-gathering events, often defined as events with greater than 1,000 people in attendance, pose significant risks for injuries and illnesses among participants. Sporting events are unique mass gatherings that have the potential to cause major public health problems, particularly when alcohol is involved. Studies have shown that college students consume significantly more alcohol on game days during the football season, which increases the incidence of high-risk behaviors, arrests, assaults, and unintended injuries.^1^–^3^ There are significant public health and safety consequences of alcohol use in the setting of mass-gathering events at both the professional and collegiate level. Studies have linked alcohol sales to increased emergency department (ED) visits at both a Marseilles, France, football stadium and a Philadelphia, PA, ballpark.^4^.^5^ In comparison, alcohol has traditionally not been sold in most major college football stadiums.

Since 2015, many schools have begun to allow alcohol sales within their football stadiums.^6^ There is a paucity of data on the public health effects of these policy changes. In 1996, the University of Colorado at Boulder banned in-stadium alcohol sales, which resulted in a dramatic decrease in arrests, assaults, ejections, and referrals to the Judicial Affairs Office.^7^ A study conducted at Ohio State University found that stricter community and university alcohol policies were associated with increased alcohol-related ED visits.^8^ Studies at the University of Iowa a few years later, however, showed that stricter alcohol policies were associated with decreased incidence of blood ethanol levels in severe intoxication range, as well as a non-significant decrease in the number of alcohol-related ED visits.^9^,^10^ Researchers at a large Midwestern university found a linear increase in alcohol-related incidents in the three years after the implementation of stadium alcohol sales.^11^ An analysis of police campus records from 12 Division 1 football universities found that criminal incidents were significantly more common on game days than non-game days, but that there was no significant increase in incidents following the introduction of in-stadium sales.^6^

Although the health impact of alcohol sales is unclear, the financial benefit to the university is more predictable. West Virginia University, for example, generated an additional $700,000 in revenue after the implementation of in-stadium alcohol sales in 2011.^12^ At the time, the university administration claimed that there was a reduction in alcohol-related game day incidents.^13^ However, later research found that alcohol-related incidents in Morgantown, West Virginia, had increased every year since the change in alcohol sales policy.^12^ The University of Texas and Ohio State University both experienced boons in revenue after starting in-stadium alcohol sales, generating in excess of $1 million.^11^ As universities increasingly turn to in-stadium alcohol sales as an additional revenue source, the health consequences of this development remain unclear.

In 2021, for the first time, alcoholic beverages were available for purchase while attending a home football game at the study location, a large Midwestern university. This stadium routinely hosts >65,000 spectators, and drinking alcohol is highly prevalent at pre-game tailgating events. This represented a sharp departure from 2010 when the university implemented a series of more restrictive alcohol policies, including tailgate party restrictions and making local bars open only to those over age 21.^9^

**Population Health Research Capsule**

What do we already know about this issue?

*Universities have recently introduced in-stadium alcohol sales at football games. The impact on local emergency medical services (EMS) utilization is unclear*.

What was the research question?

*Did the addition of alcohol sales at a college football stadium increase the incidence of alcohol-related EMS calls and emergency department (ED) visits?*

What was the major finding of the study?

*Introduction of alcohol sales resulted in no significant change in EMS calls (aOR 0.54; 95% CI 0.15–2.03), or ED visits (aOR 0.98; 95% CI 0.70–1.38)*.

How does this improve population health?

*Our evidence shows that in-stadium alcohol sales can be introduced to large sporting events on a college campus without adversely affecting public health*.

Our aim in this study was to determine the impact of this policy change on the local healthcare and emergency medical services (EMS) systems for alcohol-related complaints. We hypothesized that the wider availability of alcohol inside the stadium would lead to an increase in patients being evaluated for alcohol-related complaints. The primary outcome measures included incidence of alcohol-related emergency calls to local EMS, and incidence of alcohol-related visits to the University of Iowa Health Care (UIHC) ED. Secondary outcome measures included acuity level of alcohol-related patient presentations, hospital length of stay, and demographic factors.

## METHODS

The study site was a college football stadium at a large Midwestern university. Medical care is provided by university physicians and nurses for first-aid care inside the stadium, and local EMS provides multiple crews of paramedic and emergency medicine technician pairs as first responders for any medical emergency. Patients who are too sick or injured to be seen at the first-aid station are transported to the nearby university hospital ED.

This was a retrospective cohort study of records from county-wide ambulance service calls for service and ED hospital records. All games played in 2021 served as the exposure group, and games played in 2019 (the last full football season before the alcohol sales started in 2021) served as the control group. The 2020 season was excluded due to COVID-related effects on game attendance. Trained extractors using predefined criteria reviewed each patient treated over a 24-hour period (7 am −7 am) on the selected Saturdays and determined whether each ED visit or call was “alcohol related” or “not alcohol related.” For example, a patient seen for an ankle sprain who was also intoxicated was considered to be an alcohol-related case. A patient seen for chest pain with no report of alcohol use was not considered to be alcohol related. We compared the number of patients and proportion of alcohol-related cases seen by local EMS and the university ED on each football Saturday in 2021 to the number in 2019.

We compared characteristics of Johnson County Ambulance Services calls and UIHC ED visits before and after the onset of stadium alcohol sales using Student’s t-test for continuous variables, a chi-square test for categorical variables, and a Mann-Whitney U test for ordinal variables. We determined the odds of an EMS call or ED visit being alcohol related using logistic regression analysis. Unadjusted and adjusted odds ratios (aOR) with 95% confidence intervals are presented. A multivariable logistic regression model was developed to adjust for covariates between the two seasons, including patient age, gender, and kickoff times. We used the Hosmer-Lemeshow goodness-of-fit statistic and McFadden’s R^2^ to assess model fit. A small *P*-value indicates a lack of fit. We used Stata version 17.0 (StataCorp LLC, College Station, TX) for all statistical analysis.

## RESULTS

There were 11 games in each season, with seven home games each year played in Kinnick Stadium in Iowa City, IA. (Games played on Fridays were excluded.) The game day characteristics between 2019 and 2021 were similar with the only differences being games decided by ≤7 points, the time of kickoff, and the outdoor temperature at kickoff (Table 1).

### Ambulance Service Calls

**Figure 1:**
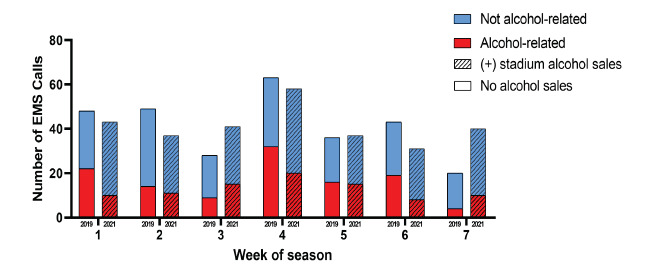
Number of emergency medical service calls on home game days, 2019 and 2021 seasons.

In 2021, after in-stadium alcohol sales began, there were a total of 505 emergency calls to local ambulance services on football Saturdays (home and away), and 29% of them were for alcohol-related incidents. This is a significant decrease from 2019, when 36% of 456 calls were alcohol related (OR 0.73, 95% CI 0.54–0.98) (Figure 1, Table 2). Looking specifically at the seven home games each season, the difference was more pronounced: 31% of calls in 2021 were alcohol related compared to 40% in 2019 (OR 0.66, 95% CI 0.47–0.93). In the first six hours after kickoff (thereby excluding calls from pre-game tailgating parties and calls many hours after the game), this reduction in calls was maintained: 27% in 2021vs 44% in 2019 (OR 0.49, 95% CI 0.28–0.86). Patients with an alcohol-related call had similar average blood-alcohol levels as measured by portable breath tests in 2021 (0.23) and 2019 (0.20) (*P*=0.46). After adjustment for covariates, the odds of a call being alcohol related were lower in 2021 than 2019, but this difference was not significant (aOR 0.83, 95% CI 0.48–1.42) (Table 2). This model appeared to be a good fit to the data (McFadden’s R^2^=0.30, *P*=0.32). Including only home games, we found that this difference was also not significant (aOR 0.54, 95% CI 0.48–1.42). The home-only model also appeared to be a good fit to the data (McFadden’s R^2^=0.30; *P*=0.86).

### Emergency Department Visits

**Figure 2:**
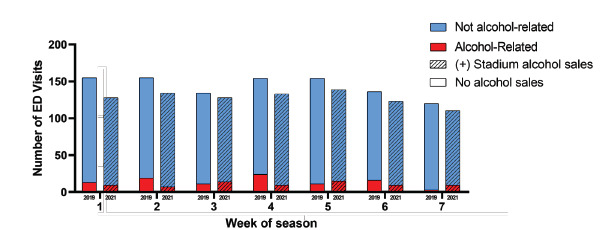
Number of emergency department (ED) visits on home game days, 2019 and 2021 seasons.

In the ED, 1,414 patients were seen on game days in 2021, 8% of them for alcohol-related reasons. This is similar to 2019, when 9% of the 1,538 patients presented due to alcohol-related complaints (OR 0.87, 95% CI 0.67–1.13) (Figure 2, Table 3). On days with a home game, the proportion of ED visits that were alcohol related was slightly lower in 2021 (8.0%) than in 2019 (9.6%), but this difference was not significant (OR 0.82, 95% CI 0.60–1.13). Looking at the six hours immediately after kickoff at a home game, however, there was a significant reduction in the rate of alcohol-related ED visits in 2021 (6.7%) compared to 2019 (13.0%) (OR 0.55, 95% CI 0.32–0.97).

There was a non-significant increase in alcohol-related visits on home (9.7%) compared to away (8.6%) game days (OR 1.13, 95% CI 0.86–1.49). None of the game factors—such as kickoff time, air temperature at kickoff, victory, rivalry game, game decided by fewer than seven points, or opponent ranked in the Associated Press Top 25 poll—were correlated with more alcohol-related visits (Table 3). After adjustment for covariates, the odds of an ED visit being alcohol-related were similar in 2021 and 2019 (aOR 0.98, 95% CI 0.70–1.38). This model appeared to be an adequate fit to the data (McFadden’s R^2^=0.21; *P*=0.88) (Table 3). Including only home games, this difference was also not significant (aOR 0.54; 95% CI 0.28–1.03). The home-only model also appeared to be an adequate fit to the data (McFadden’s R^2^=0.24; *P*=0.89). Including only home games 0–6 hours after kickoff, this difference was also not significant (aOR 0.40, 95% CI 0.15–1.09). The model also appeared to be an adequate fit to the data (McFadden’s R^2^=0.2; *P*=0.69).

## DISCUSSION

At a large Midwestern university, the onset of in-stadium alcohol sales in 2021 was not associated with an increase in alcohol-related emergencies when compared to the most recent full season of football games in 2019. In-stadium alcohol sales had no significant impact on the frequency or proportion of alcohol-related EMS calls or ED visits. There was a decrease in alcohol-related ED visits within six hours following kickoff. There was a 50% decrease in alcohol-related EMS calls and ED visits in the six hours following kickoff, despite the availability of alcohol for sale inside the stadium. As a result, we reject our hypothesis that the increased access to in-stadium alcohol would lead to more incidents. The reason for this decrease is unclear, but it is possible that fans drank less at tailgate parties knowing they could continue to consume alcohol once the game started. Yet long lines and a two-beverage limit at stadium concessions likely kept most patrons from consuming excessively.

Previous studies showed that game characteristics, such as later kickoff times, higher opponent rankings, and in-state or conference rivalry games, were associated with increased numbers of alcohol-related ejections.^14^ This suggests that there is a link between the importance of a game and misconduct/unhealthy behavior. Interestingly, our data demonstrated no such association between game characteristics and alcohol-related ED visits. Perhaps the factors of long lines and two-beverage limits at the stadium modulated the influence of game characteristics on the negative effects of alcohol consumption. Investigation of both the social and public health effects of in-stadium alcohol sales would be an interesting avenue for a future study to further evaluate this contrast in results.

Unsurprisingly, patient factors that were significantly associated with alcohol-related ED visits included male gender, age 18–30, leaving against medical advice/eloping, and discharge to police custody. This is consistent with prior data showing that men between the ages of 21–29 years are most likely to be intoxicated at college football games.^8^,^10^ While the demographics of all those attending sporting events should be included for public awareness campaigns on responsible alcohol consumption, special care should be taken by universities in conjunction with public health experts to target this particular demographic. Given the recent increase in alcohol-related ED visits among college students in general, it is important that steps are taken to reduce the morbidity associated with high-risk alcohol consumption at mass-gathering events; and young males contribute significantly to that morbidity.^15^

The policy implications of this data are important for university administrators. Considering increasing costs for athletic departments across the country, many universities are seeking additional revenue sources. Our data suggests that in-stadium alcohol sales could potentially be a revenue source without worsening negative public health consequences that are classically associated with alcohol consumption at mass-gathering events. These results also indicate that, as has been found in prior research, tailgating may contribute more to excess alcohol consumption than in-stadium alcohol sales.^16^ Thus, universities should continue to evaluate implementation of policies that would restrict alcohol consumption at tailgates as a potential route for reducing the social and public health consequences of alcohol-related risky behavior.

From a public health perspective, our study does not suggest that in-stadium alcohol sales increase the burden on local EDs and EMS agencies. Contrary to the assumption that implementing alcohol sales in mass-gathering venues will require additional EMS, medical, and police resources, it is possible that responsibly managed sales may lessen the public health burden while providing a financial benefit to the institution.

## LIMITATIONS

There are several limitations of this study. One includes the relatively limited sample size. While there was an abundance of EMS calls and ED visits within the dates studied, it will be important to analyze longitudinal trends, as was done over a three-year period in Barry’s 2019 study.^11^ Another limitation of our study is the effect of the COVID-19 pandemic. While 2020 was excluded due to the effects of the pandemic, it is possible that the 2021 data was also affected by the personal and institutional responses to the pandemic. Mask mandates were still in place, and some patrons were probably less likely to attend and consume alcohol at mass-gathering events than in prior years. However, it is important to note that there was no significant difference in overall attendance at the study site between 2019 and 2021.

It is also possible that some patients were missed by collecting only data from one ED, as the study site is in a town with two hospitals. However, the data was collected from the ED immediately adjacent to the stadium, which likely sees most if not all of the ED presentations from people attending the football games. Regardless, the EMS data was collected county-wide and, therefore, would include transports of patients taken to both EDs.

## CONCLUSION

This study represents a novel contribution to the currently limited data on the public health effects of the implementation of alcohol sales inside large, college football stadiums. There is significant morbidity associated with mass-gathering events, which is only exacerbated when alcohol becomes involved. As more schools join the trend of starting in-stadium alcohol sales, it will be increasingly important to evaluate the public health and social consequences of these policy changes. Our data suggests that there is no significant increase in the number of alcohol-related emergencies after one university’s implementation of alcohol sales. We intend to continue to collect local data annually to better inform our understanding of the risks and benefits associated with in-stadium alcohol sales in college football.

## Figures and Tables

**Table 1:** Game day characteristics of the pre- and post-stadium alcohol sales periods, 2019 and 2021 football seasons at one Midwestern university.

	No alcohol sales (2019, N=11 games)		Stadium alcohol sales (2021, N=11 games)	

Game characteristics	n	(%)	n	(%)
Home games	7	63.6	7	63.6
Victories	8	72.7	8	72.7
Games decided by ≤ 7 points	6	54.5	3	27.3
Kickoff time				
Morning	6	54.5	1	9.1
Afternoon	3	27.3	9	81.8
Evening	2	18.2	1	9.1
Kickoff temperature (°F)				
31–44	4	36.4	2	18.2
45–59	3	27.3	4	36.4
60–74	4	36.4	2	18.2
≥75	0	0.0	3	27.3
Rivalry games	3	27.3	3	27.3
Ranked inside AP top 25 poll				
Iowa Hawkeyes	11	100.0	11	100.0
Opponents	4	36.4	4	36.4

*°F*, degrees Fahrenheit; *AP*, Associated Press.

**Table 2:** Odds of an emergency medical services call being alcohol related by patient and game characteristics, college football game days, 2019 + 2021.

	Odds ratio (95% CI)	Adjusted odds ratio (95% CI)
Alcohol sales		
All games		
No alcohol sales	1.0 (ref)	1.0 (ref)
(+) alcohol sales	0.73 (0.54–0.98)	0.83 (0.48–1.42)
Home games only		
No alcohol sales	1.0 (ref)	1.0 (ref)
(+) alcohol sales	0.66 (0.47–0.93)	0.54 (0.15–2.03)
Patient characteristics		
Age		
≤17	0.23 (0.06–0.82)	0.35 (0.09–1.38)
18–20	8.72 (4.38–17.4)	7.32 (3.22–16.68)
21–30	3.52 (1.88–6.6)	3.06 (1.48–6.28)
31–40	1.07 (0.54–2.11)	1.16 (0.54–2.51)
41–50	1.0 (ref)	1.0 (ref)
51–60	1.59 (0.78–3.24)	1.91 (0.84–4.35)
61+	0.33 (0.17–0.65)	0.49 (0.23–1.03)
Gender		
Male	1.89 (1.40–2.56)	1.78 (1.20–2.63)
Female	1.0 (ref)	1.0 (ref)
Game characteristics		
Kickoff time		
Morning	1.0 (ref)	1.0 (ref)
Afternoon	1.12 (0.78–1.60)	1.52 (0.75–3.05)
Evening	1.93 (1.27–2.93)	2.02 (1.06–3.85)
Location		
Home	1.77 (1.26–2.49)	1.18 (0.65–2.16)
Away	1.0 (ref)	1.0 (ref)
Victory		
Yes	0.68 (0.48–0.95)	0.75 (0.51–1.03)
No	1.0 (ref)	1.0 (ref)

* Adjusted odds ratios obtained via multivariable logistic regression model.

*CI*, confidence interval; *ref*, reference.

**Table 3:** Odds of an emergency department visit being alcohol-related by patient and game characteristics, college football game days, 2019 + 2021

	Odds ratio (95% CI)	Adjusted odds ratio (95% CI)
Alcohol sales		
All games		
No alcohol sales	1.0 (ref)	1.0 (ref)
(+) alcohol sales	0.87 (0.67–1.13)	0.98 (0.70–1.38)
Home games only		
No alcohol sales	1.0 (ref)	1.0 (ref)
(+) alcohol sales	0.82 (0.60–1.13)	0.54 (0.28–1.03)
Visit 0–6 hours after kickoff, home only		
No alcohol sales	1.0 (ref)	1.0 (ref)
(+) alcohol sales	0.55 (0.32–0.97)	0.40 (0.15–1.09)
Patient characteristics		
Age		
≤17	0.04 (0.01–0.18)	0.04 (0.01–0.16)
18–20	4.35 (2.68–7.07)	3.95 (2.38–6.56)
21–30	2.09 (1.31–3.33)	2.06 (1.27–3.34)
31–40	1.15 (0.69–1.92)	1.02 (0.60–1.74)
41–50	1.0 (ref)	1.0 (ref)
51–60	0.93 (0.54–1.62)	0.87 (0.49–1.53)
61+	0.20 (0.10–0.40)	0.20 (0.10–0.40)
Sex		
Male	2.44 (1.84–3.23)	2.71 (2.00–3.66)
Female	1.0 (ref)	1.0 (ref)
Game characteristics		
Kick off time		
Morning	1.0 (ref)	1.0 (ref)
Afternoon	0.92 (0.69–1.24)	1.20 (0.81–1.78)
Evening	1.05 (0.73–1.51)	1.71 (1.08–2.71)
Location		
Home	1.13 (0.86–1.49)	1.23 (0.90–1.68)
Away	1.0 (ref)	1.0 (ref)
Victory		
Yes	0.77 (0.58–1.03)	0.73 (0.51–1.03)
No	1.0 (ref)	1.0 (ref)

* Adjusted odds ratios obtained via multivariable logistic regression model.

*CI*, confidence interval; *ref*, reference.
